# Computational Evolution of Beta-2-Microglobulin Binding Peptides for Nanopatterned Surface Sensors

**DOI:** 10.3390/ijms22020812

**Published:** 2021-01-15

**Authors:** Abimbola Feyisara Adedeji Olulana, Miguel A. Soler, Martina Lotteri, Hendrik Vondracek, Loredana Casalis, Daniela Marasco, Matteo Castronovo, Sara Fortuna

**Affiliations:** 1 Department of Medical and Biological Sciences, University of Udine, 33100 Udine, Italy; miguel.soler@iit.it (M.A.S.); M.Castronovo@leeds.ac.uk (M.C.); 2 Department of Physics, PhD School of Nanotechnology, University of Trieste, 34127 Trieste, Italy; 3 Regional Referral Centre for Rare Diseases, Azienda Sanitaria Universitaria Integrata di Udine, 33100 Udine, Italy; 4 School of Food Science and Nutrition, University of Leeds, Leeds LS2 9JT, UK; 5 Italian Institute of Technology (IIT), Via Melen 83, B Block, 16152 Genova, Italy; 6 Nanoinnovation Lab, Elettra-Sincrotone S.C.p.A., ss 14 km 163,5 in AREA Science Park, 34149 Trieste, Italy; martina.lotteri@gmail.com (M.L.); hendrik.vondracek@elettra.eu (H.V.); loredana.casalis@elettra.eu (L.C.); 7 Department of Chemical and Pharmaceutical Sciences, University of Trieste, Via L. Giorgieri 1, 34127 Trieste, Italy; 8 Department of Pharmacy, University of Naples “Federico II”, Via Montesano, 80134 Naples, Italy; daniela.marasco@unina.it

**Keywords:** peptides, beta-2-Microglobulin, DNA, atomic force microscopy (AFM), computational design, self-assembly, biosensor

## Abstract

The bottom-up design of smart nanodevices largely depends on the accuracy by which each of the inherent nanometric components can be functionally designed with predictive methods. Here, we present a rationally designed, self-assembled nanochip capable of capturing a target protein by means of pre-selected binding sites. The sensing elements comprise computationally evolved peptides, designed to target an arbitrarily selected binding site on the surface of beta-2-Microglobulin (β2m), a globular protein that lacks well-defined pockets. The nanopatterned surface was generated by an atomic force microscopy (AFM)-based, tip force-driven nanolithography technique termed nanografting to construct laterally confined self-assembled nanopatches of single stranded (ss)DNA. These were subsequently associated with an ssDNA–peptide conjugate by means of DNA-directed immobilization, therefore allowing control of the peptide’s spatial orientation. We characterized the sensitivity of such peptide-containing systems against β2m in solution by means of AFM-based differential topographic imaging and surface plasmon resonance (SPR) spectroscopy. Our results show that the confined peptides are capable of specifically capturing β2m from the surface–liquid interface with micromolar affinity, hence providing a viable proof-of-concept for our approach to peptide design.

## 1. Introduction

Self-assembled responsive devices for biosensing, drug delivery, and enzymes immobilization rely on the rational design of a number of functional components. A supporting surface, functionalization and immobilization strategies, and a probe capable of detecting the expected changes in its microenvironment are generally concertedly designed and arranged in a bottom-up approach [1,2,3,4,5].

**Figure 1:**
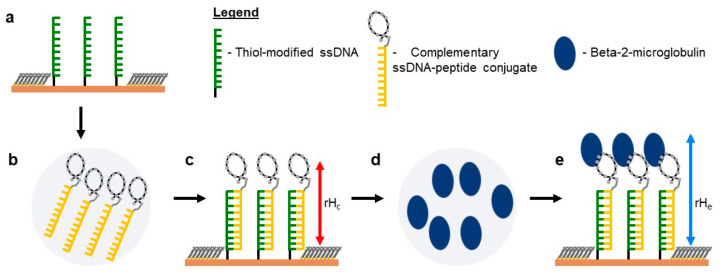
Step-by-step immobilization of dsDNA–peptide conjugate via DNA-directed immobilization (DDI) and atomic force microscopy (AFM) tip-induced nanografting. (**a**) Laterally confined thiol-modified ssDNA molecules surrounded with a bioresistant top-oligo-ethylene glycol (TOEG) monolayer. (**b**) A solution of complementary ssDNA–peptide conjugate. (**c**) The outcome of (**a**) treated with (**b**) with a measured relative height rHc (highlighted by a red arrow). (**d**) A solution of Beta-2-microglobulin. (**e**) The outcome of (**c**) treated with (**d**) with a relative height rHe (highlighted by a blue arrow). This cartoon representation does not reflect the real alignment of the molecules within the nanopatch.

For surface-bound biosensing applications, ligands as peptides can be immobilized on different surfaces such as polymeric materials, cellulose, glass, silicon wafer, and flat gold film via different immobilization approaches [6]. The peptide immobilization step is a key factor influencing recognition efficiency in peptide arrays. Several aspects can affect such an efficiency: the random adsorption of peptides, their instability, and desorption resulting in short lifetimes and heterogeneous peptide orientation on surfaces, as well as low immobilization yields and loss of bioactivity after surface immobilization. This brings us to combining DNA-directed immobilization (DDI), which allows for synergistic combination of the exquisite self-assembly ability of DNA, with the virtually unlimited functionalities of peptide design, aiming to form multifaceted semi-synthetic DNA conjugates for broad application in biosensors and theragnostics [6,7]. The concept of DDI, developed by Niemeyer’s group [8], takes advantage of Watson–Crick base pairing to immobilize DNA–protein conjugates onto a prior thiolated DNA-complementary matrix on a solid support (Figure 1a–c). Some of the advantages of DDI over automated deposition techniques are provision of a chemically mild process for parallel immobilization of the protein without instability or denaturation of the protein, reversibility and site selectivity, and spatial orientation without perturbing the bio-activity of the protein [6]. This method in conjunction with the atomic force microscopy (AFM)-based nanolithography approach has grown popular in recent years with successful applications in generating antibody arrays on an ultra-flat gold surface [9,10] and aptamer-based arrays [11]. The bioactive peptide array can then be employed for the detection or the oriented immobilization of larger molecules (Figure 1d,e).

The proposed method relies on identification of a suitable sensing element for a given target, which typically involves ligand screening techniques. Most established methods involve a phage display library [12,13,14] or, if a target protein is known, bioinformatic derivation of a minimal, ligand binding-competent protein sub-domain [15]. Both techniques are limited in their versatility in choosing a target binding site [16], which is of importance when a defined site should be selected. 

Molecular modelling has the potential to swiftly solve the issue of targeting a chosen protein site once the target structure is known [17,18,19,20]. In particular, our recently developed code [21] (recently implemented in python [22]) allows for computational evolving of a random amino acid sequence to select peptides with predicted high affinity towards a chosen target. Previous proof-of-concept work involved decapeptide design against efavirenz, an antiretroviral medication used for the treatment and prevention of HIV/AIDS [23], and chemotherapeutic drug camptothecin-11 (CPT-11) for therapeutic drug monitoring applications [24]. The latter case resulted in micromolar affinity as measured by means of surface plasmon resonance (SPR) and fluorescence spectroscopy [24]. In addition, PARCE has then been successfully used by our group for designing binders for the maltose binding protein pocket [19], for a surface-exposed site of the beta-2-microglobulin (β2m) [18], as well as by others, for the elongated adipose tissue-specific secretory factor resistin [25].

Here, we report advances in both experimental and computational techniques aimed toward peptide optimization against a specific protein site and application of AFM-based protein binding assays. This work follows our previous work on peptide design for β2m [18], a thoroughly characterized protein [26,27], dosed to determine ovarian cancer aggressiveness [28], and also known to accumulate in patients undergoing hemodialysis, eventually leading to amyloidosis [29]. Unlike previous studies, in order to challenge our bottom-up, computational approach, β2m-binding peptide design was carried by choosing a β2m-target site associated with unavailable knowledge of hitherto existing molecules binding it. On the experimental side, the selected peptides were bound to a nanochip on an ultra-flat gold substrate by means of nanografting [18,30] and DDI [8,31], whereas subsequent, differential AFM height measurements of the engineered surface allowed for detection of β2m protein capture from solution. 

## 2. Results and Discussion

### 2.1. Biding Site Selection

To identify possible peptide binding sites on the β2m surface, we employed the PeptiMap webserver [32], which is designed to map peptide binding sites when only the receptor structure is known. The tool considers the specific features of putative peptide binding and uses a Fast Fourier Transform-based docking protocol adapted for peptide–protein interaction [33]. We evaluated the putative binding sites of β2m in 12 different poses sampled along a 20 ns molecular dynamics (MD) trajectory of the free protein. From each pose, we obtained six possible binding sites (Figure S1), ranked by the number of non-bonded contacts. 

**Figure 2:**
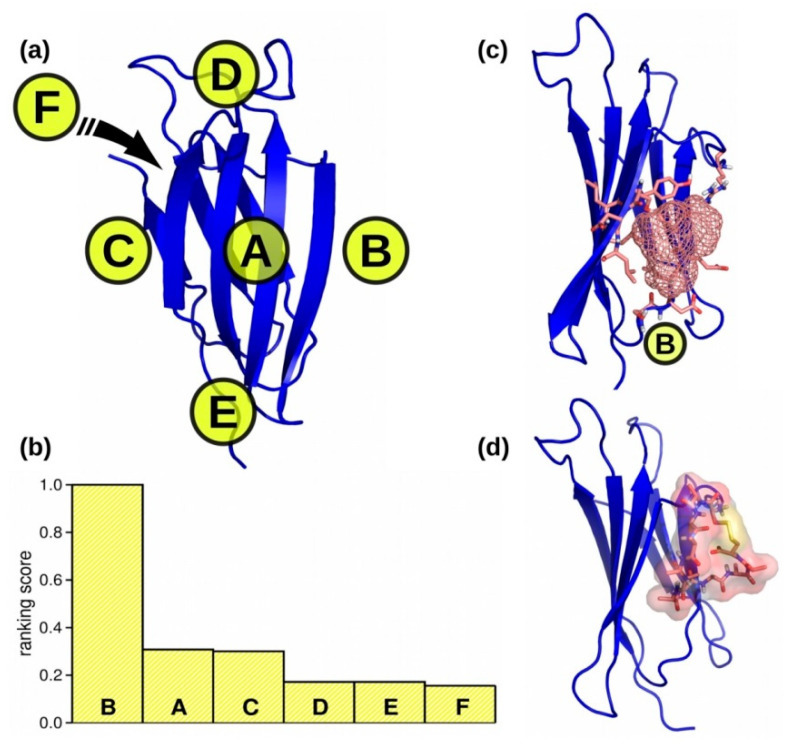
**(a**) Definition of the different regions in β2m for the classification of the binding sites identified by PeptiMap, each region is identified by a capital letter, (**b**) averaged binding sites ranking scores for each region as labelled in panel a, and (**c**) the β2m region B formed by the residues S34, D35, I36, E37, V38, R46, S53, D54, L55, L65, and Y67. (**d**) Starting structure of the cyclic dodecapeptide polyalanine bound to the β2m region B.

Each putative binding site was classified according to its location (A, B, C, D, E, and F) in the protein structure (Figure 2a). A global ranking score (R) for each location was defined as

$$R_{Z}=\frac{1}{\sum_{i=1}^{N}r_{Z}^{high}\left(i\right)},\;Z=A,B,\ldots ,F$$

where $r_{Z}^{high}\left(i\right)$ is the highest rank of any binding site located in the region *Z* in the pose *i*, and *N* is the total number of poses (*N* = 12). The calculated ranking scores (Figure 2b) show clearly that binding sites located in the region B are the most probable, since they ranked first in all poses. In this region, located between the two β-sheets of the protein (Figure 2c), we found the binding sites with the highest number of interacting residues and with the largest solvent accessible surface area (SASA) of around 900 Å^2^. We call this site “B”. Site B’s size is compatible with the previously employed 12-residue cyclic peptides, of which surface size ranges 1000–2000 Å^2^. Region A (Figure S1), ranked second, was the one chosen in our previous work [18]. We chose the binding site in region B as novel binding site for the design of a second set of peptides. 

### 2.2. Peptides Generation

As the solvent-accessible area of the chosen site B was compatible with that of a 12-residue cyclic peptide, we employed as a starting peptide a cyclic dodecapeptide Cys1-polyalanine-Cys12, which was placed close to the β2m region B (Figure 2d). We employed a planar configuration of the peptide cyclic backbone and we oriented the peptide surface in perpendicular to the protein β-sheets to guarantee the interaction of all peptide residues with the β2m region B. Subsequently, we performed energy minimization of the complex structure in vacuo to avoid any clashes between peptide and protein. PARCE [21] was then employed to evolve the peptide sequence and conformation. 

In more detail, the protocol of PARCE iteratively: (i) selects a random amino acid from the binder interacting region, mutates it, and minimizes the resulting configuration; (ii) equilibrates the system and samples the peptide–protein conformational space; (iii) estimates the binding affinity of the peptide/protein complex; (iv) accepts the attempted mutation by following the Metropolis criteria:

$$P_{\mathrm{acc}}=\min[1,\exp[-\left(E_{\mathrm{new}}-E_{\mathrm{old}}\right)/T_{\mathrm{MC}}]]$$

where $E_{\mathrm{old}}$ is the estimated binding affinity of the old configuration, $E_{\mathrm{new}}$ is that of the attempted mutation, and $T_{\mathrm{MC}}$ is a tuneable parameter for the Metropolis acceptance probability $P_{\mathrm{acc}}$. In this work, we performed the conformational sampling and estimated the binding affinity by (i) running short replica-exchange MD (REMD) to provide a conformational sampling, (ii) clustering REMD conformations with the Daura algorithm [34], (iii) scoring the representative cluster conformations by employing the Autodock Vina scoring function, and, finally, (iv) by selecting the lowest scoring representative conformation as the new configuration and the inherent score as the estimated binding affinity for the peptide/protein complex [18].

**Figure 3:**
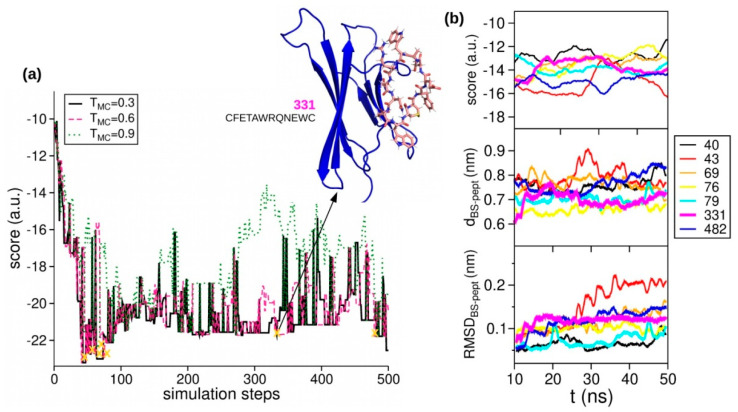
Computational peptide design. (**a**) PARCE optimization path: the optimization was performed by employing three paths with acceptance threshold $T_{\mathrm{MC}}$ = 0.3 (black), 0.6 (magenta) 0.9 (green). Peptide mutants that underwent an in silico screening phase are indicated (yellow crosses). In the inset: structure of the peptide pep331, eventually selected for AFM experiments, binding β2m region B. (**b**) Computational screening of selected peptides by analyzing three descriptors (binding score, distance between peptide and protein binding site, and RMSD) along the MD trajectories of peptide/target complexes.

The optimization (Figure 2) was carried out in parallel at three values of $T_{\mathrm{MC}}$ (0.3, 0.6, and 0.9 a.u.). Random exchanges among replicas were accepted by using the standard parallel tempering scheme [35]. As expected, the scores for the lowest $T_{\mathrm{MC}}$ decrease along the optimization path, and frequent exchanges are observed (Figure 3a). A set of low-scoring peptides predicted to have “Good water solubility” [36] was selected from each optimization path and fed forward to subsequent computational screening.

### 2.3. Peptides Screening

The generated peptides were screened by means of full solvent MD simulations to identify the optimum binder.

Specifically, 20 different soluble peptide sequences were selected from the optimization path (Figure 3a) and their MD trajectories were analyzed by using three descriptors: (i) predicted binding energy computed as Autodock Vina score; (ii) distance between the center of mass of β2m site B, and that of the peptide; and (iii) the root mean square deviation (RMSD) of the backbones of both β2m site B and peptide. According to our previous work [18,19], Vina energies higher than −10 a.u. can be associated with weak bindings between the protein and the peptide. In Figure 3b, we show the evolution of the three analyzed descriptors for the best peptide candidates, while a more detailed elaboration of the screening analysis is presented in the Supplementary Material (see Section S2, Figures S2–S4, and Table S1).

**Figure 4:**
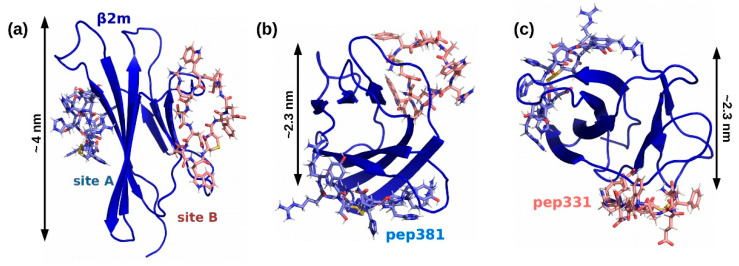
Overlap between peptides/β2m configurations as obtained by the design algorithm: (**a**) side and (**b**,**c**) top views. Color code: β2m (blue), peptide pep381 designed for site A (cyan), and peptide pep331 designed for site B (pink). Both peptides are cyclic thanks to an intramolecular S-S bridge (yellow). β2m size along selected directions is also indicated.

Several peptides, such as pep79, pep331, or pep482, show optimal behaviors for all three descriptors. In turn, final selection lies in the structural analysis of their binding conformation within the complex formed with β2m. Accordingly, we rejected sequences (i) whose binding has been significantly shifted from the region B; (ii) whose cys–cys pair is directly interacting with β2m residues, as the N-terminal should be exclusively involved in peptide–DNA conjugation for nanochip fabrication. According to these criteria, we selected the sequence CFETAWRQNEWC, which will be referred to as pep331, designed against binding region B (Figure 4, and Table 1). We compare this peptide with the formerly generated pep382 designed against the binding region A [17,18]. Of note, both peptide sequences lack Lys residues, thus leading to a unique site for DNA conjugation.

### 2.4. Affinity Measures

**Figure 5:**
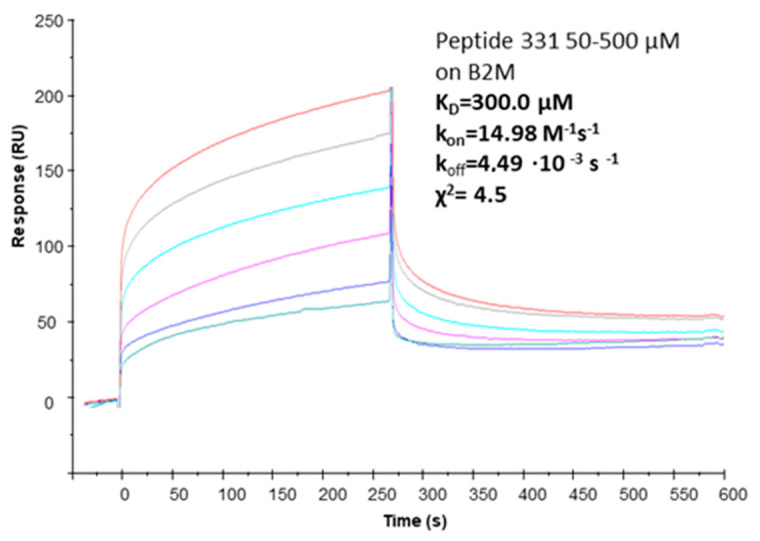
Overlay of SPR sensorgrams for the interaction of peptide pep331 on immobilized ß2M *K*_D_ = 300.0 µM, k_on_ = 14.98 M^−1^ s^−1^, k_off_ = 4.49∙10^−3^ s^−1^, χ^2^ = 4.5.

SPR assays were carried out to characterize the β2m-binding ability of pep331. In Figure 5, the sensorgram overlays are reported: the peptide exhibited a dose–response variation of RU intensity versus peptide concentrations. Kinetic parameters allowed for estimating of the dissociation constants (*K*_D_) evaluated with the 1:1 Langmuir, leading to micromolar *K*_D_ estimated values, in line with previous studies [17,18,19].

### 2.5. Self-Assembly of Functional dsDNA–peptide Nanopatch

Our strategy to immobilize the peptide on an ultra-flat gold surface requires both nanografting and DDI [9,10,11]. To obtain bioactive peptide arrays that result from the transformation of DNA arrays via hybridization with the complementary ssDNA–peptide conjugates, it becomes important to (i) use highly robust conjugation chemistry to create ssDNA–peptide conjugates, and (ii) generate DNA arrays with optimum surface density, thus ensuring steric accessibility of arrayed DNA molecules, an essential requirement for allowing further recognition reactions.

**Figure 6:**
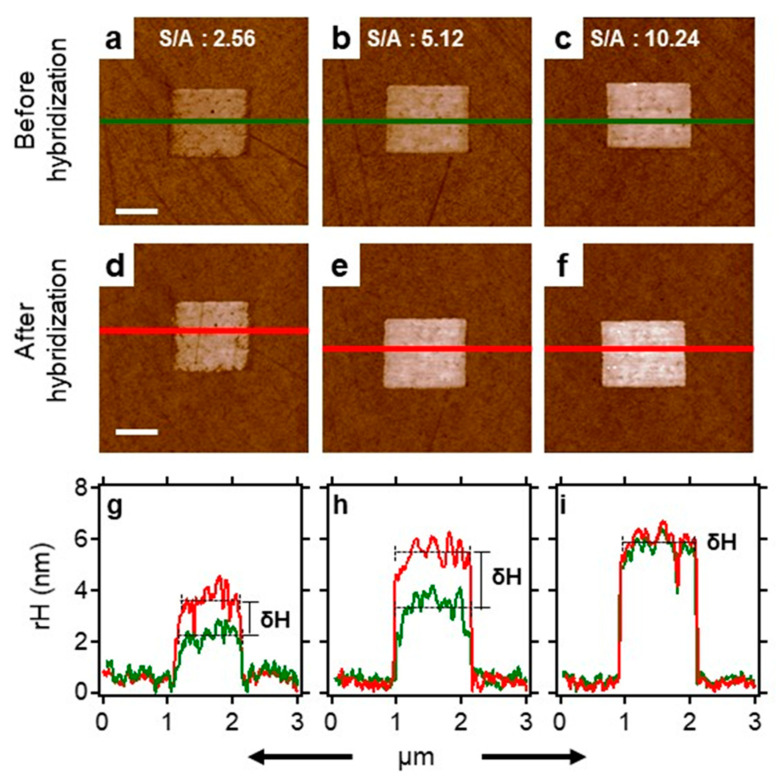
Height profile measurement of a density-dependent ssDNA nanopatch before and after treatment with 500 nM Scheme 381. conjugate. (**a**–**c**) show the AFM micrographs of a thiol-modified ssDNA nanopatch at different ssDNA densities as defined by density factor (S/A, as defined in Section 2.6). (**d**–**f**) display the AFM micrographs of the dsDNA–pep381 nanopatch obtained after treating (**a**–**c**) with the 500 nM solution of complementary ssDNA–pep381 conjugate. (**g**–**i**) show the height profiles across (**a**–**c**) and (**d**–**f**) in green and red, respectively. δH is the change in relative height corresponding to the difference between the average relative heights. These averages are highlighted as black dashed lines across the profiles. The scale bar is 600 nm for all the micrographs.

To create functional dsDNA–peptide nanopatches, we firstly verified the reactivity of the ssDNA nanopatch to its complementary ssDNA–peptide conjugate under standard hybridization conditions. Using nanografting, we created laterally confined ssDNA nanopatches (see Figure 1a) and then transformed them into a dsDNA–peptide nanopatch (see Figure 1c) by incubation with a known concentration of complementary ssDNA–peptide conjugate. The latter process hereafter is referred to as DDI. The outcomes of these two processes are shown in Figure 6a–f, respectively. Figure 6a shows the AFM image of a low-density ssDNA nanopatch before hybridization, and Figure 6d shows the same nanopatch after hybridization with 500 nM solution of complementary ssDNA–pep381 conjugate. We quantified the reactivity of the ssDNA nanopatches by measuring the relative height of the nanopatch with respect to the surrounding reference surface before and after hybridization, as shown in Figure 6g–i. The height increases (δH) in Figure 6g,h show that the ssDNA molecules within two nanopatches at low density hybridize with the complementary conjugates at the surface–liquid interface, confirming that the nanografting process can be optimized to allow hybridization reactions with the confined ssDNA molecules [37,38]. Likewise, Figure S5 (see Supplementary Material) shows an example of ssDNA nanopatches within the same density range (see S/A definition in Section 2.6) of those shown in Figure 6, but treated with 200 nM concentration of complementary solution of ssDNA–pep381 conjugate. Comparing Figure 6 and Figure S5, the δH in Figure 6g,h changes from 1.4 ± 0.2 to 2.0 ± 0.2 nm, whereas the δH for the corresponding patches in Figure S5g,h changes from 1 ± 0.2 to 1.2 ± 0.2 nm. These findings suggest that at relatively low surface density (S/A approx. between 1 and 6), there is a linear correlation between ssDNA nanopatch surface density (S/A), concentration of the complementary ssDNA–pep381 solution, and the measured topographic height change (δH). However, no height change can be observed at S/A = 10.24 (see Figure 6c,f, and Figure S5c,f, respectively) upon hybridization at any of the two investigated concentrations of complementary solution of the ssDNA–pep381 conjugate, as shown in Figure 6i and Figure S5i.

### 2.6. Surface Density Controls the Hybridization Efficiency of ssDNA Nanopatch

**Figure 7:**
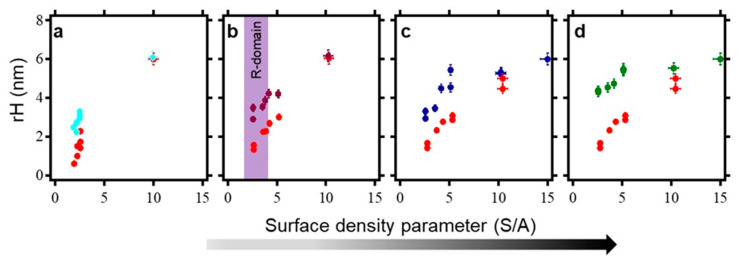
Dependency of hybridization on surface density of ssDNA nanopatch. (**a**) shows the plot of relative heights of density-dependent ssDNA nanopatches before (red) and after (cyan) treatment with 100 nM solution of complementary ssDNA–pep381 conjugate. (**b**) displays relative heights of density-dependent ssDNA nanopatches before (red) and after (plum) treated with a 200 nM solution of complementary ssDNA–pep381 conjugate. (**c**) shows the correlation between the initial relative heights of density-dependent ssDNA nanopatches (red) and the final relative heights (blue) of the same nanopatches after treated with 500 nM solution of complementary ssDNA–pep381 conjugate. Lastly, (**d**) displays the correlation of the initial relative heights (red) of density-dependent ssDNA nanopatches and the final relative heights (deep green) of the same nanopatches after treated with 1000 nM solution of the complementary ssDNA–pep381 conjugate. The R-domain is the optimum density domain at which we detect ß2m recognition. The dark bar shows the density gradient.

Systematic measurements were carried out quantify the effect of ssDNA surface density on nanopatch hybridization with the complementary ssDNA–peptide conjugate, as to determine baseline values for subsequent analysis of ß2m binding on dsDNA–peptide nanopatches. To address this, using the same AFM tip-induced self-assembly process as mentioned above, we self-assembled ssDNA nanopatches of different surface densities, exploiting the versatility of the technique that allows for fine-tuning the surface density of biomolecules chemisorbed on the gold surface during the rapid, AFM tip-induced deposition process [39]. The density-regulating parameters typically are: (i) the concentration of the biomolecule to immobilize a solution of thiol-modified ssDNA molecules (sequences in Supplementary Section S3), (ii) the applied load, and (iii) the number of times the AFM tip strokes (S) over a selected area (A) [31,38]. In this work, the concentration of the thiol-modified ssDNA molecules and applied grafting force were kept constant while the number of times that the tip stroked (thus over-writing) the preselected area was varied. The scanning-dependent, surface density parameter S/A is defined as follows:

$$\frac{S}{A}\;=\frac{R\;\times N}{W}$$

where *R* is the radius of curvature of the AFM tip in contact with surface during the nanografting process, *N* is the number of times the AFM tip strokes the selected area, and *W* is the width of the nanopatch in µm. In our experimental section, *R* and *W* were kept constant since we used only one type of AFM cantilever, i.e., NSC 18/no Al, which has ~10 nm as the radius of curvature in contact with the surface, and all the patches generated have a width of 1 µm. The only parameter that was varied is *N*, which defines the pixel lines. The master panel of the AFM workspace allows *N* to vary from 96 to 1536, as the AFM tip moves from one addressable area to another on the bio-repellent surface during the nanografting. We thus generated ssDNA nanopatches of varying surface coverage (S/A ranging from 1 to 15), followed by the acquisition of AFM topographic images. Subsequently each patch is hybridized with a known concentration of complementary ssDNA–peptide conjugates at 37 °C for 1 h. This is followed by the acquisition of AFM topographic images of the same nanopatches with inherent varying surface density, thus comprised of dsDNA–peptide molecules, allowing for comparison of height profiles before and after the hybridization (see Figure 7). In general, the plots in Figure 7 show that the relative height of the ssDNA nanopatches increases with respect to the concentration of the conjugates used for hybridization, only within the value of S/A = 1–6. Afterwards, the relative height reaches a plateau, where the relative height change between before and after the hybridization is negligible, regardless of the ssDNA–peptide concentration in solution. Based on these findings, we assumed that the hybridization is effective at the low S/A values (low density), while at high S/A values (high density), it is difficult to quantify a hybridization efficiency via AFM topographic height measurements. However, it has been established in the literature [40] that the hybridization efficiency at very high density is ~50%, as revealed by AFM compressibility measurements and theoretical modelling. With these results, it is logical to work with an optimum density that is far from the saturation plateau shown in Figure 7, towards ensuring β2m accessibility to immobilized peptide. As such, we chose surface density (S/A) of 2.5 as the optimum value for hybridization. This optimum density value is highlighted by the R-domain highlighted in Figure 7b.

### 2.7. Recognition of β2m by dsDNA–peptide Conjugate Nanopatches

**Figure 8:**
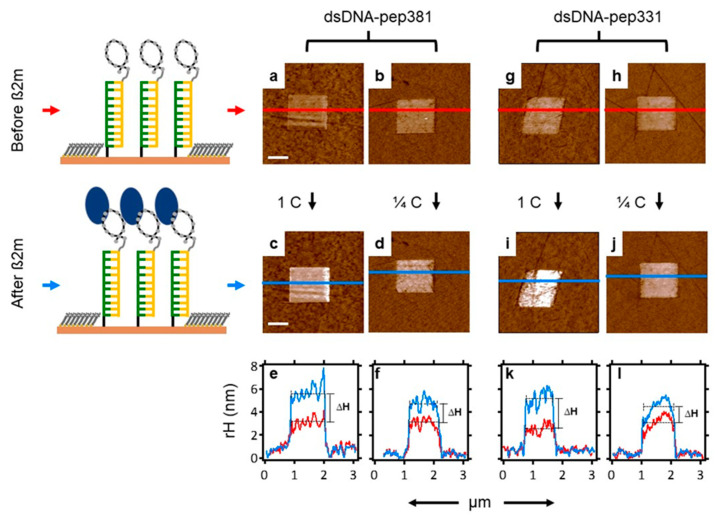
AFM detection and quantification of the recognition of ß2m by dsDNA–peptide at different concentrations of the analyte. From the left-hand side, (**a**,**b**) show the AFM images of dsDNA–pep381 before treatment with ß2m solution. On the left of (**a**), we have the schematic that depicts the laterally confined dsDNA–peptide molecules surrounded with the bioresistant TOEG monolayer. (**c**,**d**) show the same AFM images in (**a**,**b**) after treatment with 1C (5 mg/mL) and ¼ C (1.25 mg/mL) solution of ß2m. On the left of (**c**), we have a representation of binding of ß2m by the laterally confined dsDNA–peptide molecules. (**e**,**f**) represent plots of relative height profiles corresponding to the lines across ((**a**,**b**), red) and ((**c**,**d**), blue), respectively. (**g**,**h**) show the AFM images of dsDNA–pep331 before treatment with ß2m solution. (**i**,**j**) show the same AFM images in (**g**,**h**) after treatment with 1C (5 mg/mL) and ¼ C (1.25 mg/mL) solution of ß2m. (**k**,**l**) represent graphs of the height profiles corresponding to the lines across ((**g**,**h**), red) and ((**i**,**j**), blue), respectively. ∆H is the differential height. Each ∆H was calculated by subtracting the average relative height of the red trace from the average relative height of the blue trace. The thin black dashed lines indicate the averages across the profiles. The scale bar is 600 nm and is applicable for all the AFM micrographs.

Once an optimum nanopatch density was identified, we designed two chips (one for each peptide of Table 1) which then served as a screening surface for β2m detection. We utilized AFM topographic height measurement to quantitatively measure the height profile across such nanopatches before and after the incubation with a known concentration of β2m as depicted in Figure 1c,e. The differential height then becomes the binding response of the nanopatch toward the β2m [10,18,41]. In particular, Figure 8 reveals the sensitivity of both dsDNA–pep381 and dsDNA–pep331 nanopatches toward 5 mg/mL (1C) and 1.25 mg/mL (1/4C) of β2m solution (Figure 8c,d,i,j). After the binding assay with 5 mg/mL of β2m, we observed an increase in the relative height of the dsDNA–pep381 and dsDNA–pep331 nanopatches, as shown in Figure 8e,k. This yields height differences (∆H) of 2.2 ± 0.3 and 2.5 ± 0.3 nm with respect to dsDNA–pep381 and dsDNA–pep331, consistent with the predicted β2m extension in the direction orthogonal to each peptide (Figure 4b,c). The binding response of the two peptide nanopatches decreases at 1.25 mg/mL of β2m (see Figure 8f,l), yielding a height difference (∆H) of 1.7 ± 0.2 and 1.1 ± 0.1 nm with respect to dsDNA–pep381 and dsDNA–pep331. From the results obtained in Figure 8, the two cyclic peptides, pep381 and pep331, recognize their respective binding sites on β2m with relatively high sensitivity.

In addition, we investigated the specificity of dsDNA–peptide towards ß2m by performing a binding assay on a pure dsDNA nanopatch. We treated the dsDNA nanopatch with the 5 mg/mL solution of ß2m. This negative control confirms that the height differences obtained in Figure 8 only occur when the dsDNA molecules are functionalized with peptide. Indeed, the results in Figure S6 show no height change of the dsDNA nanopatch upon treatment with ß2m solution, thus providing a proof-of-concept for binding specificity.

### 2.8. Competition Assay

**Figure 9:**
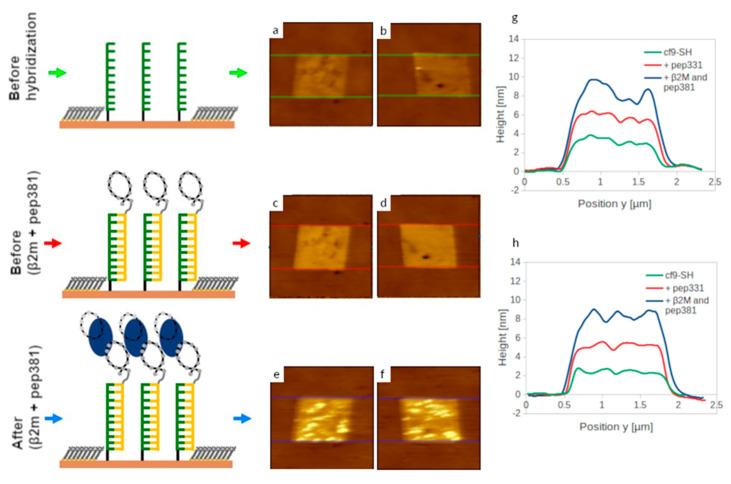
AFM detection of the ß2m/pep381 complex by dsDNA–pep331. From top to bottom: (**a**,**b**) AFM images of the ssDNA before hybridization as depicted in the left schematic from (**a**); (**c**,**d**) the same patches after incubation with 100 nM solution of the complementary ssDNA–pep331. The schematic to the left of (**c**) depicts the laterally confined dsDNA–pep331 molecules surrounded by the bioresistant TOEG monolayer. (**e**,**f**) show the same patches after incubation with the solution of the ß2m/pep381 complex and after an additional washing with TE buffer. The schematic to the left depicts the binding of the ß2m–pep381 complex by the laterally confined dsDNA–pep331 molecules. (**g**) Heights profiles of patches (**a**,**c**,**e**) averaged over the area between the corresponding lines. (**h**) Height profiles of patches (**b**,**d**,**f**) determined as in (**g**).

The AFM measurements suggest that both peptides are capable of capturing β2m; however, the patch height upon binding β2m is very similar, as expected (Figure 4b,c). To confirm that the two designed peptides bind to two different sites of the same protein, we performed an AFM-based competition assay. The AFM setup was the same as that employed in the previous section by immobilizing pep331 as described in the former section (Figure 9a–d), and the nanopatches were incubated with a test solution containing the β2m/pep381 complex. As the test solution was prepared by mixing β2m with a 10-fold molar excess of pep381, to move the equilibrium towards the complex formation, we expected the complex to be adsorbed on the pep331 monolayer only if its binding site on β2m was not covered by pep381. Indeed, after incubation, the imaged patches showed an increase in height and the formation of aggregates (see Figure 9e,f). This is also evident when considering the surface roughness, which was found to increase from 1.09 to 2.79 nm after incubation with the β2m/pep381 complex. In order to take this non-homogeneous structure into account, the height of the patches was determined by averaging over the entire patch. Doing so, a ∆H = 2.00 +/− 0.46 nm (1.25 +/− 0.42 nm after further washing) has been determined. This is in the same range as the height differences obtained in the recognition assay (Figure 8) and confirms the recognition of β2m by pep331 also in the presence of pep381, suggesting the two peptides bind to different binding sites of the β2m.

When we attempted to invert the experimental setup by immobilizing pep381 and by employing the β2m/pep331 complex for incubation, we observed the precipitation of the complex and accurate height measurements could not be performed.

## 3. Materials and Methods

### 3.1. Binding Site Selection

The β2m structure with PDB code 1LDS [42] was first minimized with the steepest descent minimization method before undergoing a 20 ns NPT molecular dynamics simulation in explicit water solvent at 300 K. The equilibration process before the production run consisted of two short 100 ps NVT + 100 ps NPT runs. Other MD parameters used in this run are similar to those further described in “Molecular Dynamics simulation”. We selected 12 representative snapshots sampled at constant time intervals from the trajectory. These configurations were analyzed by PeptiMap [32], which identifies six possible binding sites on the protein surface suitable for peptide binding. We activated the PPI mode, a minor modification to the scoring function which reduces the weight of a cavity term and thus optimizes the results on a PPI test set.

### 3.2. Peptide Design

Peptides were designed by using a modified version of PARCE.sh (version 2019), formerly known as BINDesignER [21], a free BASH wrapper that can be found here: https://github.com/PARCE-project/PARCE.sh, accessed on 14 January 2021), and also available in Python. The simulation design employed AutoDock Vina [43] as the scoring function, the program tleap in AmberTools12 [44] to perform the amino acid mutations, and Gromacs v.4.5.5 [45] to carry out the minimization, replica exchange molecular dynamics (REMD) simulations, and clustering analysis. Minimizations and REMDs were carried out by employing the AMBER99SB-ILDN [46] force field. Optimizations were run without solvent to optimize the speed of the design simulation. After each mutation, the system was energetically relaxed by performing three successive minimizations: (i) a partial minimization only for the side chain of the mutated residue, (ii) a partial minimization for the mutated and both nearest neighboring residues, and (iii) a global minimization of the system. Subsequently, a REMD simulation of the relaxed system was carried out by using 8 different NVT replicas at T = 375, 391, 407, 423, 440, 458, 477, and 495 K. The backbone of the β2m was restrained by using a harmonic potential of force constant 1000 kJ mol^−1^ nm^−2^. All bonds were restrained by using the LINCS algorithm [47]. The time length of each replica was 1 ns, while the time step was 2 fs and the replica exchange step every 2 ps. Peptide-β2m poses were printed out every 2 ps. We clustered the peptide-protein poses obtained from all replicas by using the Daura method [34], as implemented in Gromacs, with a cut-off of 0.105 nm. We considered only clusters containing more than 10 structures for the scoring evaluation. The solubility of the accepted peptide mutants was assessed with the Innovagen peptide calculator webtool [36] to identify those with “Good water solubility”.

### 3.3. Molecular Dynamics Simulations

Selected β2m/peptide complexes then underwent 50 ns NPT MD simulations in water with 0.15 M of NaCl, using the AMBER99SB-ILDN [46] force field along with the tip3p water model, all implemented in the Gromacs 4.5.5 molecular package [45]. We used the leapfrog Verlet integrator with a time step of 1 fs. The h-bonds were constrained by using the LINCS algorithm. The temperature was established at 300 K and controlled with a modified Berendsen thermostat [48], while the pressure with an isotropic Parrinello–Rahman was at 1 bar. The equilibration protocol of the β2m/peptide system before the production run is divided in 5 stages, 4 NVT and 1 NPT, in order to heat the system gradually, avoiding unrealistic high perturbations to the binding of the complex: (i) First NVT equilibration of 25 ps, with Β2M+peptide frozen, and water and ions at 100 K; (ii) Second NVT equilibration of 50 ps, with Β2M+peptide at 200 K, and the water and ions at 100 K; (iii) Third NVT equilibration of 50 ps, with Β2M+peptide at 300 K, and the water and ions at 200 K; (iv) Fourth NVT equilibration of 100 ps, with the system at 300 K; (v) NPT equilibration of 200 ps, with the system at 300 K and 1 atm. Along MD simulations, β2m-peptide geometries were printed out every 250 ps, obtaining in total 200 structures, and were subsequently used to calculate the Binding Energy using the binding score of Vina [43]. Additionally, the h-bond and hydrophobic contacts of each β2m–peptide complex at 0, 25, and 50 ns were analyzed using the LIGPLOT tool [49] to identify the residues of the binding site in every complex. Besides Vina binding energy, all descriptors used in this report were calculated using the tools implemented in the Gromacs 4.5.5 molecular package [45].

### 3.4. Materials

Two distinct sequence thiol-derivatized (with C_6_ linker) single stranded (ss) DNA and their corresponding complementary DNA (with amino-C_6_-link) were purchased at HPLC-purified grade from Biomers GmbH (Ulm, Germany). Top-oligo-ethylene glycol (TOEG 6 (1-mercaptoundec-11-yl) hexa(ethyleneglycol), (HS-((CH_2_)_11_)-(O-CH_2_-CH_2_)_6_ -OH)) was purchased from Prochimia Surfaces (Gdynia, Poland); sodium chloride (NaCl), Tris-EDTA, sodium phosphate dibasic (NaH_2_PO_4_), sodium phosphate monobasic (NaHPO_4_), and absolute ethanol (99.8%) were all purchased from Sigma Aldrich (Milano, Italy). All buffer solution was prepared in ultra-pure water (miliQ-H_2_O) of resistivity 18.2 MΩ·cm at 25 °C and filtered before use with a sterile syringe filter (of 0.22µm pore size). The cyclic peptides were purchased at 95% purity from ProteoGenix SAS (Schiltigheim, France). In addition, Beta-2-Microglobulin (Human species) was purchased from Sigma Aldrich (Germany).

### 3.5. Surface Plasmon Resonance

The interaction between the protein and computationally optimized peptides was measured using the BIAcore 3000 (GE Healthcare, Milano, Italy). β2m was immobilized at a concentration of 100 μg/mL in 10 mM acetate buffer pH 5 (flow rate 5 μL/min, time injection 7 min) on a CM5 Biacore sensor chip [50], using EDC/NHS chemistry following the manufacturer’s instructions. Residual reactive groups were deactivated by treatment with 1 M ethanolamine hydrochloride, pH 8.5. The reference channel was activated with EDC/NHS and deactivated with ethanolamine. The binding assays were carried out at 20 μL/min at 25 °C, with 4.5 min contact-time. Peptides were diluted in the HBS running buffer (10 mM Hepes, 150 mM NaCl, 3 mM EDTA, pH 7.4). Analyte injections were performed at the indicated concentrations. The sensor surface was regenerated by using 10mM NaOH for 1 min. The association phase (k_on_) was followed for 250 s, whereas the dissociation phase (k_off_) was followed for 300 s. The instrument BIAevaluation analysis package (version 4.1, GE Healthcare, Milano, Italy) was used to subtract the signal of the reference channel.

### 3.6. ssDNA–peptide Conjugates Preparation

SoluLink’s superior bioconjugation method was exploited to prepare peptide–oligonucleotide conjugates in three steps. (i) Peptide modification: a volume that represents 10 mole equivalents of HyNic/mole protein was added to the peptide (concentrated 2.0 mg/mL) and mixed. The reaction was carried out at room temperature for 1.5 h. (ii) Oligonucleotide modification: the oligonucleotide ssDNA-amine (Biomers GmbH, Ulm, Germany) was desalted into nuclease free water using a 5K MWCO VivaSpin diafiltration apparatus and OD/µL concentration at 260 nm was determined (0.4 OD/μL). A volume containing 20 equivalents S-4FB was added to the oligonucleotide solution and incubated at room temperature for 2 h. Next, the 4FB-modified oligonucleotide was desalted into conjugation buffer (100 mM phosphate, 150 mM NaCl, at pH 6.0). (iii) DNA–peptide conjugation: taking into account the concentration and the mass of the HyNic-modified peptide to be functionalized and concentration and MSR of 4FB-modified oligonucleotide, volumes of the two components to be mixed are determined; 1/10 volume 10× TurboLink Catalyst Buffer (100 mM aniline, 100 mM phosphate, 150 mM NaCl, pH 6.0) was added to the conjugation solution and the reaction was carried out overnight at 4 °C. The concentration of the ssDNA–peptide conjugates was determined spectrophotometrically by the absorbance at A354 due to the formation of the chromophoric conjugate bond. The aforementioned conjugation method was used for the two cyclic peptides, resulting into ssDNA–pep381 and ssDNA–pep331 conjugates, where the cD1 sequence was used for the former and the cD2 sequence was used for the latter.

### 3.7. Preparation of Ultra-Flat Gold Substrate

Ultra-flat gold substrates were prepared as described in past publications from our group [31,37,51]. Briefly, a sequential deposition of gold was employed using electron beam evaporation. Firstly, gold was deposited at the rate 0.05 nms^−1^ until a film of 5 nm was obtained, then the rate of evaporation was increased to 0.1 nms^−1^ until a film of 100 nm thick was formed on freshly cleaved mica (Mica New York Corp., New York, NY, USA), clear ruby muscovite) at 10^−6^ mbar, at room temperature but in an ultra-high vacuum environment. The planar gold sheet of (111) crystallographic plane obtained on mica was sliced into a few millimeter squares (approximately 5 × 5 mm^2^) in area using Stanley-199 blade. To transfer the ultra-flat gold surface from mica to the polished side of smaller square (smaller than sliced gold on a mica sheet) p-doped silicon wafers, a drop of SU-8 photoresist adhesive (negative tone photoresist, MicroChem, Round Rock, TX, USA) was evenly dispensed on the polished side of the silicon. Then, a sandwiched square was obtained of silicon-gold-mica by impressing the polished section of silicon on the gold section of mica. All silicon-gold-mica sandwich squares were cured at 130 °C for at least 24 h. The samples were allowed to cool down to room temperature without any external cooling system and this was performed to avoid thermal stresses that can result into gold film detachment from the mica substrate. Without any further surface treatment, the samples were stored at room temperature, ready to be used for self-assembled monolayer preparation.

### 3.8. Preparation of Top-Oligo-Ethylene-Glycol SAM (TOEGSAM) on the Ultra-Flat Gold Substrate

An ultra-flat gold surface was obtained from the silicon-gold-mica sandwich by mechanical stripping of the mica substrate from the silicon-gold-mica sandwich. This was immersed and incubated in the solution of 100 µM of top-oligo-ethylene glycol (TOEG, 6, (HS-((CH_2_)_11_)-(O-CH_2_-CH_2_)_6_-OH), Prochimia and Sigma Aldrich) in absolute ethanol and 1M NaCl, TE1X (10 mM Tris-HCl, 1 mM EDTA, pH 7.2 at 25 °C in Milli-Q water) for 6 h. The TOEG template then serves as bioresistant for aspecific adsorption of macromolecules on the surface. After the incubation time, serial rinsing was performed in both ethanol and 1M NaCl, Te1X buffer to remove any physically adsorbed contaminants.

### 3.9. Nanografting of Thiol-Modified ssDNA in Contact Mode

Firstly, TOEGSAM passivated gold substrate was adhered firmly on a home-made liquid cell. The ssDNA solution (1 µM ssDNA, IM NaCl, TE1X) was evenly dispensed on the SAM and it was transferred onto the AFM X-Y scanner stage. Secondly, we performed preliminary scanning in tapping mode at low force to obtain the topographic image of the TOEGSAM surface. This allowed us to have a survey of the surface and select a flat and clean section for nanografting. At high force (≈120 nN), 1 × 1 µm^2^ was selected for the grafting process; the prior alkanethiols were exchanged for thiol-modified ssDNA molecules in solution. After grafting process, a 20 × 20 µm^2^ section that contains the grafted patches was scanned at very low force (high set point) in tapping mode.

### 3.10. Hybridization with Complementary ssDNA–Peptide Conjugates

After the nanografting process, the sample was thoroughly rinsed with 1M NaCl, TE1X buffer (DNA-free buffer), followed by a hybridization procedure with 200 nM of ssDNA–peptide conjugates solution in 1 M NaCl, Te1X at pH 7.2 and the hybridization process took place at 37 °C for 1 h. This was repeated for different concentrations of the ssDNA–pep381 so as to obtain the plots in Figure 7. Next, the sample was copiously rinsed with 1M NaCl, TE1X buffer (DNA-free buffer). This was to remove any un-bound molecules and other excess molecules that were not part of the hybridization. At the end of hybridization process before β2m binding assays, we performed AFM topographic imaging at low force (~0.2 nN) in contact mode and acquired AFM micrographs of all the nanografted nanoassemblages that had undergone hybridization. This imaging section allowed us to compare the AFM micrographs of ssDNA nanopatches to the same after hybridization with ssDNA–peptide conjugates and measure their respective relative heights.

### 3.11. Beta-2-Microglobulin Binding Assay

The dsDNA–peptide nanoassemblages were incubated with the known concentration of β2m solution for a period of 1 h at 25 °C. Subsequently, dsDNA–peptide nanoassemblages were imaged at low force (~0.2 nN) in contact mode and height topographic images were acquired. Next, we measured the relative height of dsDNA–peptide assemblage after the binding assay using the section tool in Igor Pro 6.3A software. The same software was used for image processing.

### 3.12. AFM Imaging of dsDNA–peptide Assemblages before and after the β2m Recognition Assay

The topographic images were acquired in contact mode with a soft probe (CSC38/no Al) at the lowest force (≈0.2 nN) within the DNA-free buffer (1M NaCl, TE1X buffer). For the topographic images after the β2m recognition assay, the images were acquired in 25 mM sodium phosphate, 50 mM NaCl, and pH 7. The latter buffer aids the β2m recognition while the former buffer is required for forming laterally confined DNA assemblages on surfaces.

### 3.13. Competition Assay

A test solution containing 0.33 mg/mL of β2m (corresponding to 30 μM) and a tenfold excess of pep381 (300 μM) was prepared. The test solution was pre-incubated for 1.5 h in 1 M NaCl, Te1X at pH 7.2 to allow the β2m/pep381 complex to form. The dsDNA–peptide nanoassemblages were then incubated with the test solution for a period of 1.5 h at 25 °C. Subsequently, the dsDNA–peptide nanoassemblages were imaged at low force (~0.2 nN) in contact mode and height topographic images were acquired. Since the formation of aggregates on top of the patches was observed, an additional wash with buffer was performed prior to recording a second image. The resulting height and roughness of the patches was determined by computing the average of 12 nanografted patches.

## 4. Conclusions

In the present study, we have integrated molecular modelling design, DDI, and nanografting to generate self-assembled, surface-arrayed dsDNA–peptide nanostructures, with controlled spatial orientation, for the detection of two distinct solvent exposed binding sites on β2m.

We have shown that computational design employing PARCE [21] allows for differentiating among several binding sites in silico. We have introduced a new procedure for selecting several putative binding sites for the protein of interest, in our case Beta-2-microglobulin. The procedure was based on a short molecular dynamics trajectory to sample the target conformational space. The short trajectory is appropriate for a small globular protein such as the one discussed here. If the target was larger or more flexible, a longer trajectory capable of accounting for larger rearrangements would have been needed for accurate sampling. Peptide sequences were effectively screened and ranked in relation to (i) their binding energies, (ii) distances between the center of mass of the β2m and the peptide, (iii) root mean square deviations of the binding site (BS) with respect to the peptides in different conformations, and (iv) the hydrophobic surface area of the peptides, using the combination of Autodock Vina, LIGPLOT, and Gromacs. Meanwhile, for this design, we employed a single scoring function [18,19], leading to peptides with micromolar SPR measured binding affinity. It is worth noticing that the optimization of large binders, such as antibody fragments, could be further optimized by integrating a collection of scoring functions, towards improved binding affinity [52,53,54].

We have conjugated each peptide to an ssDNA strand and applied AFM nanopatterning to construct a laterally confined self-assembled peptide monolayer by DDI on a surface. The assembly process ensured peptide spatial orientation control and allowed for system optimization towards detection of β2m in solution by means of AFM-based differential topographic imaging, by varying several self-assembly parameters.

Our results show the confined peptides are capable of specifically capturing β2m from the surface–liquid interface with micromolar affinity. This proof-of-concept work paves the way to the next challenge, which will be designing more complex devices in the form of coupled, or multivalent, binders [55,56,57,58] or antibody fragments [52,59] capable of further enhancing the binding affinity of the designed architectures.

## Figures and Tables

**Table 1:** Selected peptides sequences and their surface plasmon resonance (SPR)-determined binding affinity (*K*_D_).

ID	Sequence	Remark	*K*_D_ (SPR)
pep381	[CRRYSHQHYRHC]	cyclic with S-S bridge	38 ± 9 µM (Ref. [18])
pep331	[CFETAWRQNEWC]	cyclic with S-S bridge	300.0 µM (χ^2^ = 4.5)

## Data Availability

The data presented in this study are available on request from the corresponding author.

## Supplementary Materials

The following are available online at https://www.mdpi.com/1422-0067/22/2/812/s1. Figure S1: Binding site analysis of the β2m. Figure S2. Evolution of the Binding Energies of the B2M-peptide complexes. Figure S3. Evolution of the distance between the center of masses of the binding site of the B2M and the peptides. Figure S4. Evolution of the RMSD between the binding site of the B2M and the peptides. Figure S5. Height profile measurement of density-dependent ssDNA nanopatch before and after treatment with 200 nM solution of complementary ssDNA-pep381 conjugate. Figure S6. dsDNA treated with Beta-2-microglobulin. Table S1. Average values and standard deviation values (in parenthesis) of descriptors used for the screening analysis of the selected B2M-peptide complexes.

## Abbreviations

ß2m	Beta-2-microglobulin
DNA	Deoxyribonucleic acid
ss	Single stranded
ds	Double stranded
AFM	Atomic force microscopy
SPR	Surface plasmon resonance
DDI	DNA-directed immobilization
MD	Molecular dynamics
REMD	Replica-exchange molecular dynamics
RMSD	Root mean square deviation
K_D_	Dissociation constant
S/A	Surface density parameter
BS	Binding site
