# Supplementary material for: Computational Evolution of Beta-2-Microglobulin Binding Peptides for Nanopatterned Surface Sensors
Source: Int J Mol Sci. 2021 Jan 15;22(2):812. doi: 10.3390/ijms22020812 (PMC7831021; doi:10.3390/ijms22020812)
Supplement: Supplementary file 1 [file ijms-22-00812-s001.pdf]

SUPPLEMENTARY INFORMATION

Computational evolution of beta-2-microglubulin binding peptides for nanopatterned surface sensors

Abimbola Feyisara Adedeji Olulana [1,2,3,4,*], Miguel A. Soler [1,5], Martina Lotteri [6], Hendrik Vondracek [6,7], Loredana Casalis [6], Daniela Marasco [8], Matteo Castronovo [1,2,3,4] and Sara Fortuna [1,7,*]

1 Department of Medical and Biological Sciences, University of Udine, 33100 Udine, Italy; miguel.soler@iit.it (M.A.S.); M.Castronovo@leeds.ac.uk (M.C.)
2 PhD School of Nanotechnology, Department of Physics, University of Trieste, 34127 Trieste, Italy
3 Regional Referral Centre for Rare Diseases, Azienda Sanitaria Universitaria Integrata di Udine, 33100 Udine, Italy
4 School of Food Science and Nutrition, University of Leeds, Leeds, LS2 9JT, UK
5 Italian Institute of Technology (IIT), Via Melen 83, B Block, 16152 Genova, Italy
6 Nanoinnovation Lab, Elettra-Sincrotone S.C.p.A., ss 14 km 163,5 in AREA Science Park, I-34149 Basovizza, Trieste, Italy; martina.lotteri@gmail.com (M.L.); loredana.casalis@elettra.eu (L.C.)
7 Department of Chemical and Pharmaceutical Sciences, University of Trieste, Via L. Giorgieri 1, 34127 Trieste, Italy; hendrik.vondracek@elettra.eu (H.V.)
8 Department of Pharmacy, University of Naples “Federico II”, Via Montesano, 4980131 Naples, Italy; daniela.marasco@unina.it (D.M.)
* Correspondence: a.adedeji@sheffield.ac.uk (A.F.A.O.), s.fortuna@units.it (S.F.)

S1. Binding site selection

(a)

time (ns)	binding site rank 1	2	3	4	5	6
0.00	B	B	B	C	B	A
1.2	B	C	A	A	C	D
2.5	B	A	D	C	A	F
3.7	B	C	E	E	C	A
5.0	B	D	A	C	C	A
7.5	B	C	A	A	C	B
10.0	B	A	E	F	D	C
11.2	B	A	A	C	C	E
12.5	B	A	C	F	A	E
15.0	B	B	C	D	A	D
17.5	B	A	E	C	A	A
20.0	B	C	A	C	A	A

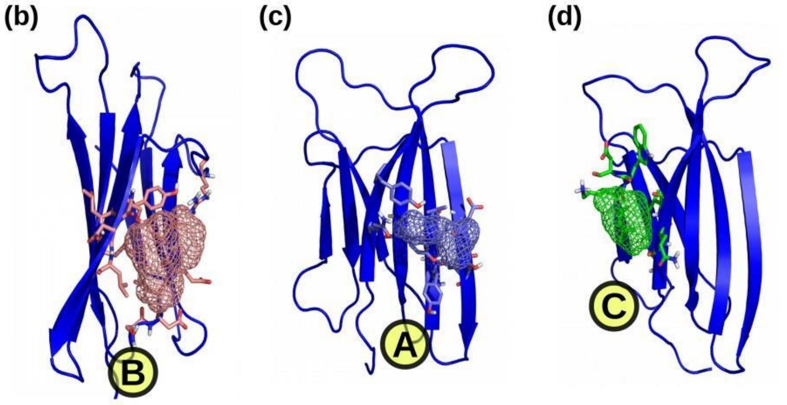

Figure S1. Binding site analysis of the β2m. (a) Ranking of the different binding sites obtained for each β2m conformation. Graphical output of the most representative β2m binding sites identified in regions (b) B, (c) A, and (d) C.

S2. Peptide screening: computational analysis

Binding Energy. In Figure S2 we show the evolution of the binding scores of the β2m-peptide complexes along the MD trajectory and in the Table S2 we included the resulting average binding scores with their standard deviation. The criteria for considering good binders with these descriptors are the low average score values as well as a stable behavior along the trajectory. According to these results, we find as best binders the peptide 43, which has the lowest average binding score, peptide 482, with the 2nd lowest average score and the lowest standard deviation value, and peptides 331 and 79, whose binding score curves show a quite stable behavior and with low values along the MD trajectory. In contrast, peptides 50f, 52, 72, 74, 84, 91, 279f, 332, 350, 359, and 460 are discarded since their binding scores are clearly above -10 a.u., which we have established as the upper limit for weak bindings.

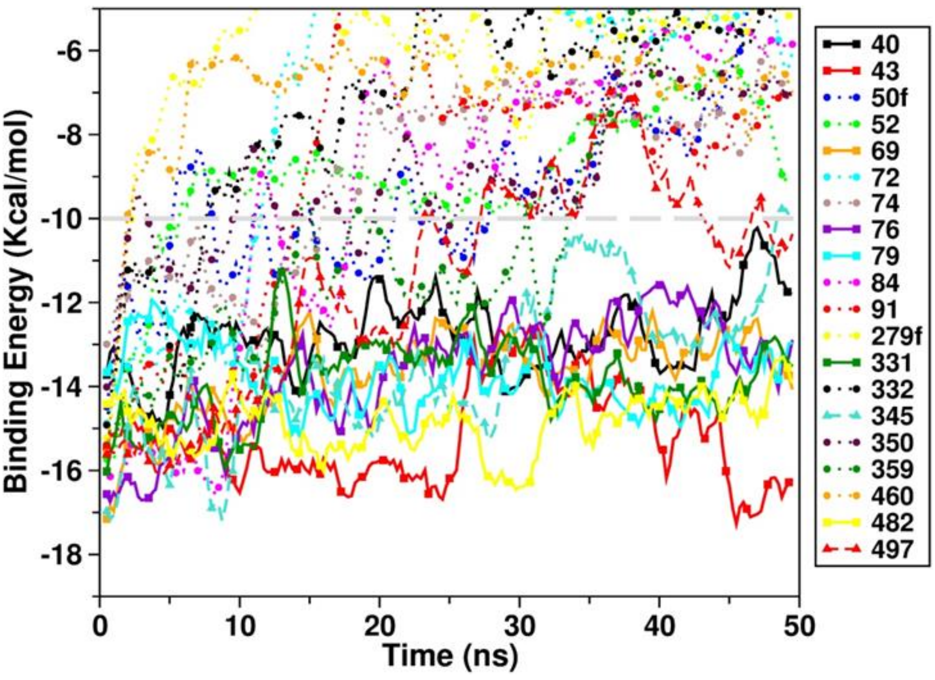

Figure S2. Evolution of the Binding Energies of the B2M-peptide complexes.

Distance B2M-peptide. Figure S3 show the evolution of the distance between the center of masses of the β2m binding site (BS) and the peptide for each complex. In this descriptor, the comparison between average distance values to select the best peptide binders is not straightforward, due to the structural differences of the designed peptide sequences and the fact that the residues in the BS are defined specifically for each peptide-protein complex according to their interactions. Nevertheless, we can consider that distances higher than 1 nm correspond to weak bindings. A better criterion to identify optimal binders is the profile of the

distance curves along the MD trajectory, i.e. the best binders are those with stable curves. Thus, we find that distance curves of peptides 76, 79, 331, 40, 43, 69, and 482 show stable behaviors (see Fig. S3). In all cases, the standard deviation value was lower than 0.05 nm.

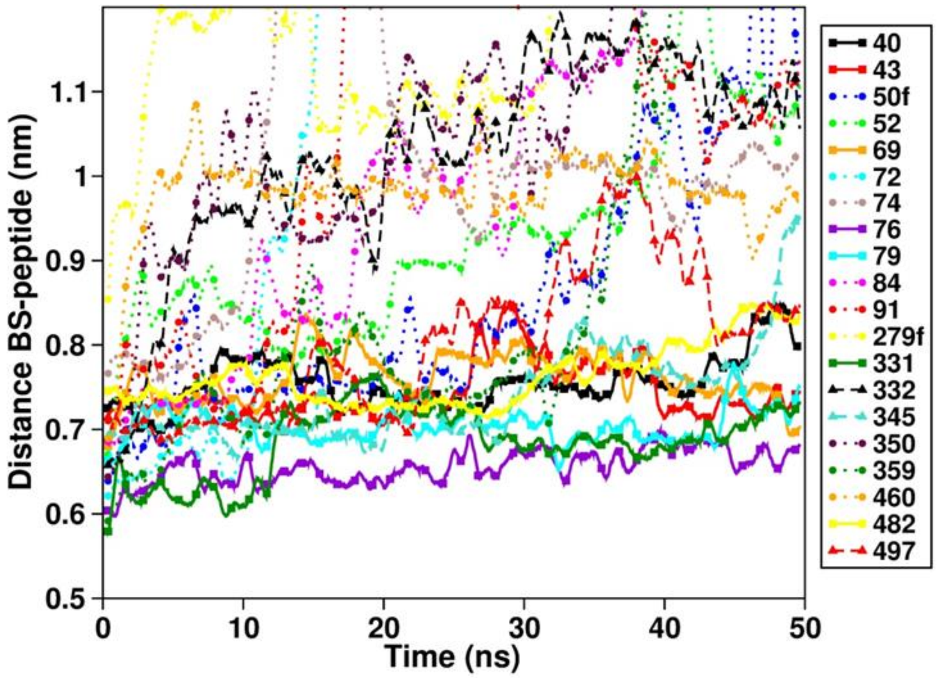

Figure S3. Evolution of the distance between the center of masses of the binding site of the B2M and the peptides.

RMSD BS-peptide backbone. In Figure S4 we show the evolution of the Root-Mean-Square Deviation (RMSD) of the BS-peptide backbone complexes along the MD trajectory. The RMSD curves show the conformational stability of the β2m-peptide complex, and if any possible conformational rearrangement occurs when the curve typically increases its value and reaches a higher plateau. Major conformational changes along the trajectory indicate low stability of the binding. The analysis of the curves is started from the geometries at 10 ns to avoid the initial structural rearrangements in the simulation caused by the equilibration of the complex in the box of explicit water solvent. We found that peptides 40, 76, 79, 331, and 460 as the best binders, with low and stable RMSD values along the trajectory (see Figure S5).

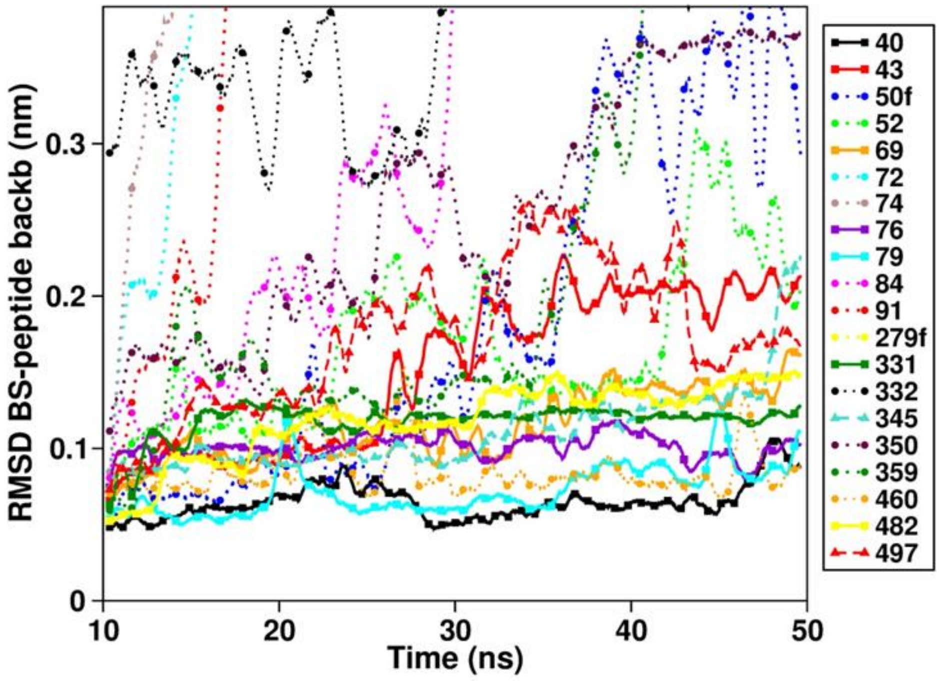

Figure S4. Evolution of the RMSD between the binding site of the B2M and the peptides.

Table S1. Average values and standard deviation values (in parenthesis) of descriptors used for the screening analysis of the selected B2M-peptide complexes.

Name	Sequence	Energy Score (kcal/mol)	BS-pept Distance (nm)	RMSD (nm^2)
pep40	CWERQFKLYGKC	-12.79(0.91)	0.75(0.03)	1.66(0.14)
pep43	CDGYWLRKAQWC	-15.19(1.15)	0.74(0.03)	2.57(0.19)
pep69	CWERQHQDYTKC	-13.68(0.95)	0.76(0.03)	1.91(0.17)
pep76	CWERQHQMYAKC	-13.66(1.32)	0.66(0.02)	2.08(0.15)
pep79	CWERQHQMYLKC	-13.75(0.73)	0.70(0.02)	2.02(0.13)
pep331	CFETAWRQNEWC	-13.85(0.93)	0.68(0.04)	1.70(0.12)
pep482	CYNRVYRKWHRC	-14.87(0.66)	0.76(0.03)	1.69(0.06)

S3. Design of distinct ssDNA sequences

We utilized Nupack DNA design software suite[1] to design two distinct 22 nt long sequences and check if there is cross hybridization between these sequences and their corresponding complementaries. These stem sequences and their complementaries are tagged D1, D2 and CD1, CD2 respectively. The complementary sequences, CD1 and CD2, were covalently conjugated with pep381 and pep331 using the solulink biojugation kit (see materials and methods section). See the sequences below:

D1 SH $(CH_2)_6$-5’-TTCGGCTCATACTCTGACTGTA-3’

cD1 amino link-C6-5’-TACAGTCAGAGTATGAGCCGAA-3’

D2 SH $(CH_2)_6$-5’-CTTATCGCTTTATGACCGGACC-3’

cD2 aminolink-C6- 5’-GGTCCGGTCATAAAGCGATAAG-3

S4. Density-dependent hybridization

In Figure S5, we show the products of nanografting process, presented as AFM micrographs of ssDNA nanopatch surrounded with the biorepellent alkyl thiol, from the lowest surface density (S/A = 2.56) to the high surface density (S/A = 10.24), before hybridizing with 200 nM solution of ssDNA-pep381 conjugate. Qualitatively, from the left to the right, the contrast of the AFM images increase towards the AFM image with S/A of 10.24, see Figure S5a-c. After hybridization, there is an increased contrast compared to before hybridization, see Figure S5d-f. Beyond the qualitative results, we employed AFM height measurements to compare the relative heights of ssDNA nanopatches to the dsDNA-pep381 nanopatches, i.e. relative height profiles of the AFM micrographs before and after hybridization. Notably, the relative height profiles (rH) in the third row of Figure S5 clearly shows that the height of the ssDNA nanopatches (green colour), increases as the surface density (S/A) increases with an incremental step from left to the right-hand side of the Figure. Likewise, the relative height profiles after the hybridization step (red colour) increases with respect to S/A = 2.56 – 5.12, but at the very high density (S/A = 10.24), the relative height profile corresponding to before and after hybridization is equivalent to one another. These results agree with the results obtained by Scoles and co-workers[2,3], even though the complementary DNA molecules used for hybridization by authors were longer and not conjugated with any protein or peptide.

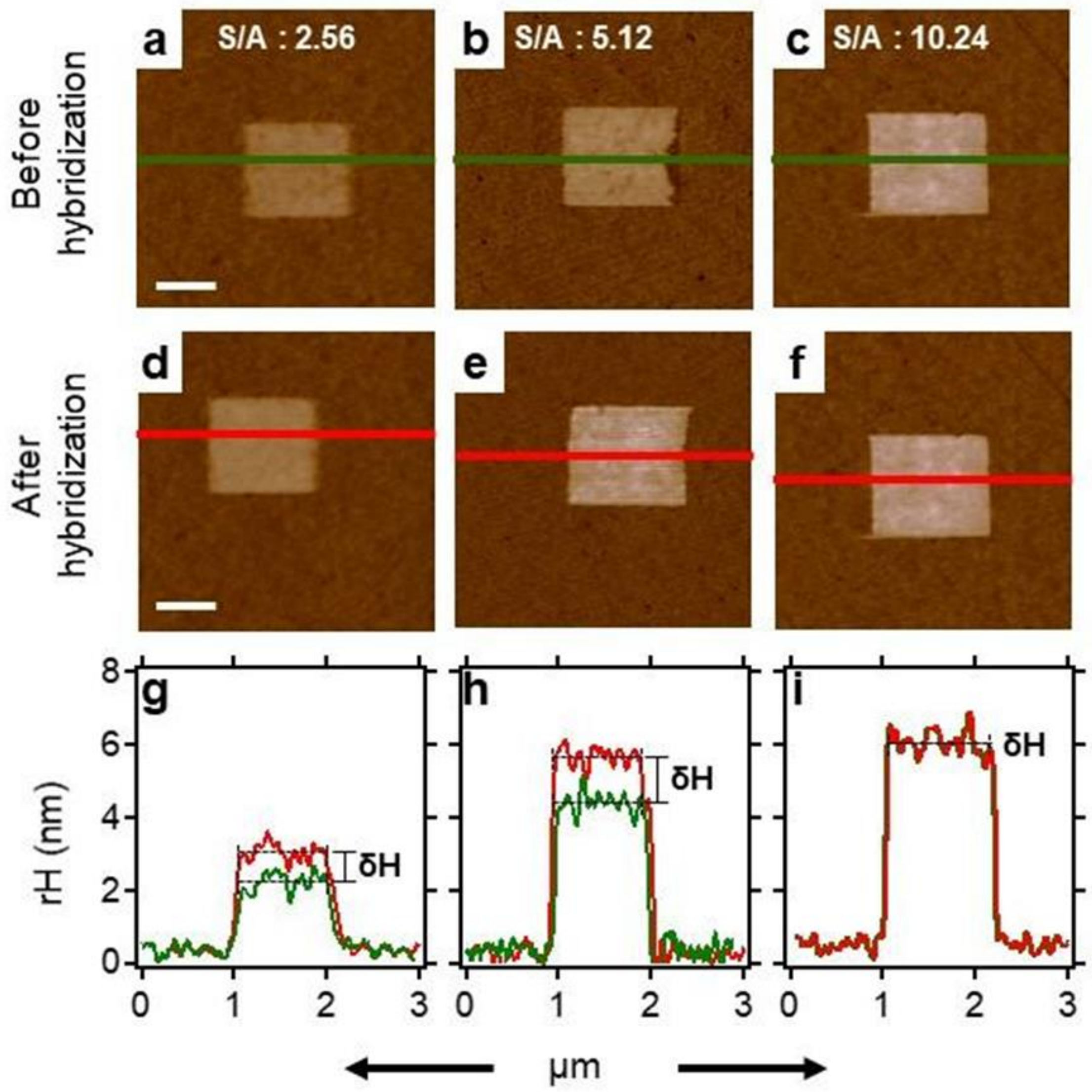

Figure S5. Height profile measurement of density-dependent ssDNA nanopatch before and after treatment with 200 nM solution of complementary ssDNA-pep381 conjugate. (a-c) Show the AFM micrographs of thiol-modified ssDNA nanopatch, constituted with different densities of ssDNA molecules as defined by density factor (S/A). (d-f) Display the AFM micrographs of dsDNA-pep381 nanopatch obtained after treating (a-c) with the 200 nM solution of complementary ssDNA-pep381 conjugate. (g-i) Show the height profiles across (a-c) and (d-f), coloured in green and red respectively. δH is the change in relative height corresponding to the difference between the average relative heights. These averages are highlighted as dash black lines across the profiles. The scale bar is 600 nm and is applicable to all the micrographs.

S5. Negative control for ß2m recognition

In Figure S6, we show that the binding of ß2m to any of the two pepetides used in this study is highly specific, as there was no binding of ß2m to dsDNA nanopatch. This further confirms that the measured ΔHs in Figure 8 are due to the presence of peptide in the nanopatch and not otherwise.

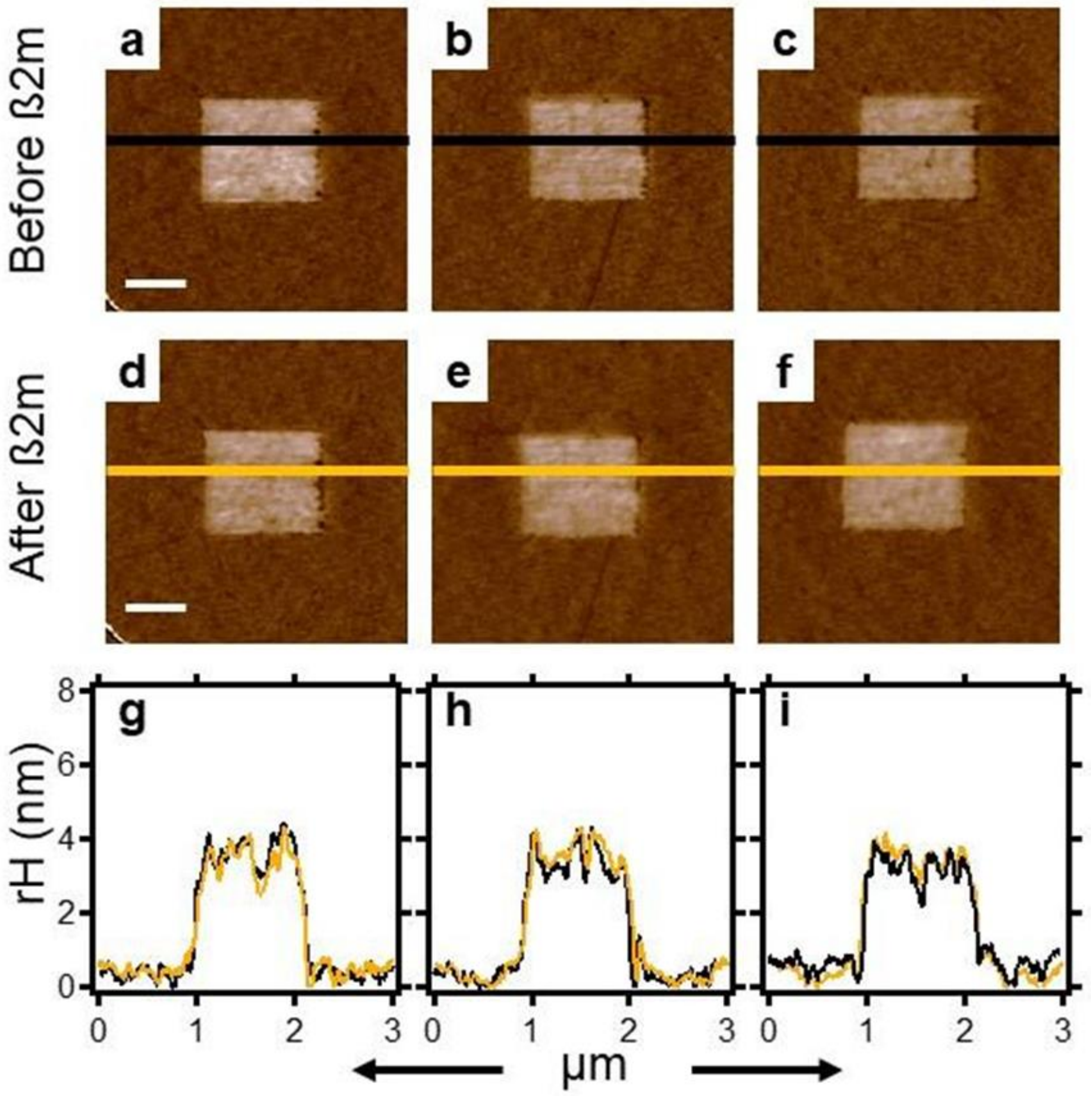

Figure S6. dsDNA treated with Beta-2-microglobulin. (a-c) Show AFM micrographs of dsDNA nanopatch with the same density. (d-f) Display the same micrographs in (a-c) after incubation with 5 mg/ml solution of Beta-2-microglobulin. (g-i) Show the relative height profiles across micrographs in the first row (a-c) and the second row (d-f), coloured in black and orange respectively. The scale bar 600 nm is applicable to all the AFM images. The result was obtained in three independent experiments.

References

1. Zadeh, J.N.; Steenberg, C.D.; Bois, J.S.; Wolfe, B.R.; Pierce, M.B.; Khan, A.R.; Dirks, R.M.; Pierce, N.A. NUPACK: Analysis and design of nucleic acid systems. *J. Comput. Chem.* **2011**, *32*, 170–173.

2. Mirmomtaz, E.; Castronovo, M.; Grunwald, C.; Bano, F.; Scaini, D.; Ensafi, A.A.; Scoles, G.; Casalis, L. Quantitative study of the effect of coverage on the hybridization efficiency of surface-bound DNA nanostructures. *Nano Lett.* **2008**, *8*, 4134–4139.

3. Castronovo, M.; Radovic, S.; Grunwald, C.; Morgante, M.; Casalis, L.; and Scoles,G. Control of Steric Hindrance on Restriction Enzyme Reactions with Surface-Bound DNA Nanostructures. *Nano Lett.* **2008**, *8*, 4140–4145.
